# Postpartum insomnia in a woman who has given birth to twins : A case report

**DOI:** 10.1192/j.eurpsy.2024.1612

**Published:** 2024-08-27

**Authors:** E. Arroyo Sánchez, P. Setién Preciados, C. Diaz Mayoral

**Affiliations:** Hospital Universitario Príncipe de Asturias, Madrid, Spain

## Abstract

**Introduction:**

Postpartum insomnia is a significant and often overlooked mental health concern affecting mothers during the postnatal period. Sleep disturbances during this critical time can have far-reaching implications for maternal well-being and the quality of care provided to newborns.

**Objectives:**

The primary objective of this review is to analyze recent clinical literature on postpartum insomnia to gain a deeper understanding of its epidemiology, clinical features, and management approaches. By synthesizing the latest research findings, this review aims to inform healthcare professionals and policymakers about the significance of postpartum insomnia and promote early recognition and intervention.

**Methods:**

A case report of a 43-year-old woman in the fifth month postpartum after a twin birth who comes to the emergency department accompanied by her partner with thoughts of death and impulse phobias due to insomnia of months of evolution. Also a systematic search of the PubMed database was conducted using the keyword “Postpartum insomnia,” and articles published between 2013 and 2023 were included. A total of 20 clinical articles meeting the inclusion criteria were analyzed to provide a comprehensive overview of postpartum insomnia.

**Results:**

The review reveals that postpartum insomnia is a prevalent and often underdiagnosed condition, affecting a significant proportion of new mothers. Risk factors such as maternal age, parity, social support, and hormonal fluctuations have been identified. Diagnostic challenges arise due to the overlap of symptoms with postpartum mood disorders, necessitating a comprehensive clinical assessment. Recent research emphasizes the importance of non-pharmacological interventions, including sleep hygiene education, cognitive-behavioral therapy for insomnia (CBT-I), and mindfulness-based approaches, as the first-line treatment options. However, pharmacotherapy may be considered in severe cases. Untreated postpartum insomnia has been associated with adverse maternal and infant outcomes, including impaired bonding, increased risk of postpartum depression, and developmental delays in infants.

**Conclusions:**

In conclusion, postpartum insomnia is a prevalent yet often underrecognized mental health concern with multifaceted clinical implications. This review highlights the importance of early detection and intervention to mitigate its impact on maternal well-being and infant development. The integration of non-pharmacological interventions, particularly CBT-I and mindfulness-based strategies, into routine postpartum care holds promise in improving sleep quality and overall postnatal mental health. Healthcare providers should be vigilant in assessing and addressing postpartum insomnia to optimize the well-being of both mothers and infants

**Disclosure of Interest:**

None Declared

